# Rationality/Anti-emotionality Personality and Dietary Habits in a Community Population in Japan

**DOI:** 10.2188/jea.JE2007421

**Published:** 2008-08-07

**Authors:** Kumi Hirokawa, Chisato Nagata, Naoyoshi Takatsuka, Natsuki Shimizu, Hiroyuki Shimizu

**Affiliations:** 1 Department of Epidemiology and Preventive Medicine, Gifu University School of Medicine

**Keywords:** Personality, Rationality, Repressed Emotion, Dietary Habits, Lifestyle

## Abstract

**Background:**

There are no strong and consistent predictors of dietary habits although some associations have been shown with psychological factors. The purpose of the present study was to examine the relationships between the rationality and anti-emotionality (R/A) personality and dietary consumption in a Japanese community.

**Methods:**

The Takayama study is a community-based cohort study on diet and cancer in Gifu, Japan, and was initiated on September 1, 1992. Cross-sectional analyses were conducted on dietary and lifestyle data. The consumption of 169 food and beverage items was measured along with portion size by using a food frequency questionnaire. Questions regarding the R/A-personality scale and lifestyle habits were included in the questionnaire. The participants were 28077 adults (13082 males and 14995 females) aged 35 years and over.

**Results:**

Both males and females with high R/A-personality scores (i.e., high degree of rational thought and emotional repression) consumed more soy products, green and yellow vegetables, other vegetables, and seaweed than the other participants. Males with high R/A-personality scores drank fewer alcoholic beverages, and females with high scores were found to snack less on sweet and salty foods than the other participants. Males with high R/A-personality scores showed higher consumption of meat and dairy products, and females with high scores showed higher consumption of fish, shellfish, and eggs than those with low R/A-personality scores.

**Conclusion:**

The R/A-personality scale may differentiate dietary habits in males and females in a Japanese community.

## INTRODUCTION

Dietary habits are related to nutritional intake and are the predictors of several diseases and health conditions, such as obesity, cardiovascular disease, cancer, and osteoporosis.^1^^-^^3^ Research on the relationship between diet and disease has frequently yielded inconclusive results due to small sample sizes and the corresponding lack of diversity with regard to previous diet and lifestyle data.^4^ Nevertheless, some general relationships between diet and disease are currently known, including those between selenium, carotenoids, and cancer; vitamin E, ω-3 fatty acids, and coronary heart disease; dietary fat and obesity; dietary sodium and hypertension; and alcohol intake and stroke.^5^^-^^11^ To prevent these chronic diseases, modification of dietary habits is often required, but this may be hindered by an individual’s food preferences, health consciousness, and other psychological factors.^12^ There are no strong and consistent predictors of dietary habits although factors such as sex, age, ethnicity, weight, mental stress, and personality characteristics show associations with dietary habits.^13^^-^^16^

The rationality and anti-emotionality (R/A) personality (blocked emotion and conscious suppression of emotion during interpersonal communication) is considered a cancer-prone personality.^17^^-^^21^ In contrast, results from Japanese studies have demonstrated an inverse association with chronic diseases, indicating that higher scores on the R/A-personality scale were associated with lower prevalence of stroke in males and females and lower prevalence of diabetes in males^22^ and with a lower risk of death from all causes in females.^23^ Thus, conflicting data exist regarding the relationship between personality traits and mortality in Western and Japanese societies. In addition, several Western and Japanese studies have reported that such personality traits are not associated with cancer risk.^24^^-^^27^ Thus, not only are the findings regarding the relationships between personality traits and chronic diseases inconsistent, but there are only a few studies that have examined whether personality traits are related to dietary habits, which are believed to be one of the risk factors for chronic diseases.

The purpose of the present study was to examine the relationships between R/A personality and diet consumption in the residents of a Japanese community. It was predicted that individuals who scored high on the R/A-personality scale would have more health-oriented dietary habits than those who scored low on this scale. For example, it was predicted that individuals with high scores would show higher consumption of vegetables and fruits and lower consumption of alcoholic beverages than those who had low scores.

## METHODS

### Participants

The Takayama study is a community-based cohort study on diet and cancer in Gifu, Japan, and was initiated on September 1, 1992. Questionnaires were distributed to all the 36990 residents of Takayama City, Gifu, Japan who were aged 35 years and over. The final response rate was 90.3% (33399/36990). After eliminating participants for whom data regarding sex, age, R/A-personality scale, medical history, and/or smoking habits were missing, questionnaires from 13082 males and 14995 females (mean ages: 54.2 and 55.1 years, respectively) were obtained. The participants classified according to their individual medical histories are as follows: 163 males and 463 females with cancer, 802 males and 758 females with cardiovascular diseases, 2679 males and 2775 females with hypertension, 824 males and 441 females with diabetes mellitus, 3149 males and 2059 females with internal diseases, and 910 males and 1427 females with allergies.

The participants were divided into 3 groups based on the R/A-personality score tertile that was used in a study by Hirokawa et al.^23^ The 0-5 group (33.7% males and 26.6% females) comprised the lower level; the 6-8 group (37.7% males and 39.2% females) comprised the middle level; and the 9-11 group (28.6% males and 34.2% females) comprised the higher level. The distribution of age, body mass index (BMI), weight, physical activity, marital status, years of education, smoking habits, and medical history by sex and the R/A-personality scores are shown in Table 1.

**Table 1.** Characteristics of participants by sex and the rationality/anti-emotionality personality group.

	Males			*P* for trend	Females			*P* for trend
	Rationality/anti-emotionality score				Rationality/anti-emotionality score			
	Lower (0-5)	Middle (6-8)	Higher (9-11)		Lower (0-5)	Middle (6-8)	Higher (9-11)	
n	4419	4950	3713		4017	5939	5039	
Age (y)	51.7	53.8	57.7	<0.01	51.9	53.8	59.3	<0.01
Body mass index (kg/m2)	22.6	22.5	22.3	<0.01	22	22	21.9	0.60
Physical activity (METs)	27.5	26.3	23.7	<0.01	16.8	18.5	17	0.97

Marital status (%)				0.03				<0.01
Without partner	9.8	8.7	8.4		24.8	23	29.2	
Married	90.2	91.3	91.6		75.2	77	70.8	

Years of education (%)				<0.01				<0.01
<5	1.1	0.4	0.7		2.8	1.1	1.6	
6-8	16.8	20.9	26.2		17.6	21.5	32.7	
9-11	38.1	33.5	32.6		37.1	41.1	39.3	
12-14	33.1	32.6	28.5		35.9	31.5	23.1	
>15	10.9	12.6	12		6.5	4.8	3.4	

Smoking* (%)				<0.01				<0.01
Non-smoker**	15.4	17.1	19		78.7	83.2	86.4	
<10 cigarettes/d	13.1	15.3	17.8		10.9	9	7.8	
11-20 cigarettes/d	36.4	35.7	38.5		8.1	6.2	4.7	
>21 cigarettes/d	35.1	31.9	24.7		2.3	1.6	1.1	

Medical history (%)								
Cancer	1.1	1.2	1.6	0.06	2.7	2.7	3.8	<0.01
Cardiovascular diseases	5.9	5.7	7.1	0.03	4.5	4.7	5.9	<0.01
Internal diseases	22.9	24.2	25.3	0.01	13	13.7	14.4	0.06
Diabetes mellitus	6.7	5.8	6.5	0.70	2.7	2.4	3.7	<0.01
Hypertension	19.4	19.9	22.5	<0.01	16.2	17.5	21.6	<0.01
Allergy	7.5	7.1	6.2	0.02	10.7	9.4	8.8	<0.01

*P* for trend shows the associations between the rationality/anti-emotionality personality group and demographic variables.

* : The number of cigarettes smoked per day was inquired among current smokers and ex-smokers.

** : Individuals who had never smoked.

### Questionnaires

The R/A-personality scale consists of 11 items (Appendix). The scale was developed by Grossarth-Maticek et al^19^^,^^20^ to assess characteristics such as rational thought and emotional repression. Participants were asked to answer either “1” for yes or “0” for no. A high score indicated a tendency for thinking rationally and repressing emotion, while a low score indicated a tendency for thinking less rationally and expressing emotion. The questions were translated into Japanese by Mizunuma et al.^28^ The internal consistency of the R/A-personality scale was tested in preceding studies.^22^^,^^23^ Cronbach’s alpha coefficients for the present study were 0.71 for both males and females.

In this cohort study, dietary consumption was assessed using a food frequency questionnaire (FFQ), and the validity and reproducibility of the FFQ have been reported.^29^ In the FFQ, 169 food and beverage items commonly used in Japan for FFQs were included along with information on portion size. The frequencies of most items were assessed using 8 response categories, ranging from never or hardly ever to 2 or more times a day over the past year. The quantities of dietary consumption were estimated in g/d for 12 arbitrarily selected food categories, which were the typical food groups consumed regularly by Japanese residents. Among the food categories, soy products were included because they are known to be associated with a reduced risk of certain cancers and cardiovascular diseases.^30^^-^^32^ For alcoholic beverages, frequencies were assessed using 9 categories, and the range was extended to 4 or more times a day over the past year. The consumption of 4 alcoholic beverages was estimated in terms of the amount (g) of pure ethanol/d. Participants who answered “never or hardly ever consume alcohol” were considered to consume 0 g pure ethanol/ d. The FFQ, like all methods of dietary assessment, is subject to measurement error. However, in the present study, the questionnaire had been validated by comparison with 12 daily diet records maintained over 1 year. Spearman’s correlation coefficients between the questionnaire and the 12 daily diet records maintained over a 1-year period for grains and potatoes, soy products, fish and shellfish, meat and meat products, eggs, milk and dairy products, green and yellow vegetables, other vegetables, fruit, snacks (sweet and salty), seaweed, and alcohol were 0.20, 0.75, 0.61, 0.18, 0.23, 0.90, 0.47, 0.66, 0.38, 0.57, 0.31, and 0.87 in males, respectively. The corresponding values in females were 0.49, 0.62, 0.33, 0.62, 0.51, 0.77, 0.53, 0.23, 0.48, 0.74, 0.53, and 0.78 respectively.^33^^-^^35^ The nutritional values of the items were obtained from the Standard Tables of Food Composition in Japan, 4th edition.^36^

In a self-administered questionnaire, age, height, weight, current marital status (“married” categorized as 1 or “without partner” categorized as 0), years of education, and major medical history (cancer, cardiovascular diseases, internal diseases, diabetes mellitus, hypertension, and allergy) were polled. Physical activity based on metabolic equivalents (METs), which measure daily energy expenditure, was assessed using the questionnaire.^37^^,^^38^ Information concerning current smoking habits was also sought. Participants were asked if they currently smoked, had stopped smoking, or had never smoked. If they reported to have ever smoked, they were asked the number of cigarettes they smoked per day.

### Statistical Analysis

Age, smoking, and total energy intake were selected as confounders based on a previous study.^39^ Medical history, which was considered to be associated with R/A personality,^22^ was also selected as a confounder. In addition, the following correlations were determined based on Spearman’s correlation coefficients (for men and women respectively) for each food consumed. BMI was significantly correlated with many food groups, for example, soy products (r = 0.03 and 0.05), green and yellow vegetables (r = 0.02 and 0.06), and other vegetables (r = 0.08 and 0.09). Physical activity was significantly correlated with soy products and green and yellow vegetables (r = 0.10 and 0.07) and other vegetables (r = 0.14 and 0.12). Years of education was also significantly correlated with soy products (r = -0.05 and -0.02) and seaweed (r = -0.05 and 0.02). These parameters were selected as confounders. To examine differences between the 3 groups classified according to the R/A-personality scores (i.e., higher, middle, and lower levels), analysis of covariance (ANCOVA) was performed, controlling for age, BMI, the number of cigarettes smoked per day, physical activity (METs), years of education, medical history, and total energy intake as covariates. Multiple comparisons using the Dunnett-Hsu test were also conducted as post hoc analyses. A *P* value less than 0.05 was used to indicate statistical significance of the results of ANCOVA. All statistical analyses were performed using the computer program PC-SAS^®^, version 6.12.^40^

## RESULTS

Table 1 shows that R/A personality was linearly associated with age, marital status, years of education, smoking, cardiovascular diseases, hypertension, and allergy in the case of both males and females. Participants in the higher level of the R/A-personality scale were older and were more likely to be non-smokers, suffer from cardiovascular diseases and hypertension, and have fewer allergies than those in the lower level. A high percentage of males in the higher level of the R/A-personality scale were married, whereas a low percentage of females in the higher level of the R/A-personality scale were married. The R/A-personality scale was also linearly associated with BMI, physical activity, and internal diseases in the case of males and with cancer and diabetes mellitus in the case of females. Males in the higher level of the R/A-personality scale had a lower level of physical activity and lower BMI and a higher percentage of internal diseases. The percentage of cancer and diabetes mellitus was higher in females in the higher level of the R/A-personality scale than in those in the lower level.

Table 2 shows the adjusted means of food consumption. Both males and females in the higher level of the R/A-personality scale showed higher consumption of soy products, green and yellow vegetables, other vegetables, and seaweed than that shown by participants in other levels of the R/A-personality scale. Males in the higher level of the R/A-personality scale consumed 6.2-13.4% more of these vegetables and seaweed than males in the lower level did, and females in the higher level consumed 7.0-10.7% more of these foods than females in the lower level did. Males in the higher level of the R/A-personality scale consumed 4.0% more meat and meat products and 6.8% more milk and dairy products than that consumed by males in the lower level. Males in the middle level of the R/A-personality scale consumed 1.5% more grain and potatoes than that consumed by males in the lower level. Females in the higher level of the R/A-personality scale consumed 4.2% more fish and shellfish and 2.9% more eggs than those in the lower level did and consumed 10.2% fewer sweet snacks and 9.0% fewer salty snacks than those in the lower level did.

**Table 2.** Adjusted mean (95% confidence interval [CI]) of food consumption (g/d) by the rationality/anti-emotionality personality group.

Item	Males										Females									
	Rationality/anti-emotionality score										Rationality/anti-emotionality score									
	Lower (0-5)	(95% CI)		Middle (6-8)	(95% CI)		Higher (9-11)	(95% CI)		P^*^	Lower (0-5)	(95% CI)		Middle (6-8)	(95% CI)		Higher (9-11)	(95% CI)		P^*^
Grains and potatoes	388	(385-390)	b	393	(391-396)	a	389	(387-392)	b	<0.01	330	(328-333)		330	(328-331)		329	(327-331)		0.73
Soy products	98	(96-100)	b	100	(98-102)	b	104	(102-106)	a	<0.01	87	(85-89)	b	93	(91-94)	a	95	(93-96)	a	<0.01
Fish and shellfish	103	(101-104)		104	(102-105)		106	(104-108)		0.05	81	(80-82)	b	84	(83-85)	a	84	(83-86)	a	<0.01
Meat and meat products	75	(73-76)	b	75	(74-76)	b	77	(76-79)	a	<0.01	61	(60-62)		62	(61-63)		61	(60-62)		0.68
Eggs	48	(47-49)		49	(48-50)		49	(48-50)		0.23	42	(41-43)	b	43	(43-44)	a	43	(43-44)	a	0.04
Milk and dairy products	189	(183-196)	b	199	(192-205)		202	(195-210)	a	0.03	229	(222-236)		231	(226-237)		235	(229-242)		0.40
Green and yellow vegetables	126	(123-129)	c	133	(130-136)	b	143	(140-147)	a	<0.01	144	(141-148)	c	155	(152-158)	b	160	(156-163)	a	<0.01
Other vegetables	225	(222-229)	c	234	(230-237)	b	243	(238-247)	a	<0.01	227	(223-230)	b	239	(236-242)	a	242	(239-246)	a	<0.01
Seaweed	23	(22-23)	b	24	(23-24)		24	(24-25)	a	<0.01	24	(23-24)	c	25	(25-26)	b	26	(26-27)	a	<0.01
Fruit	118	(114-121)		121	(118-125)		124	(120-128)		0.06	133	(130-137)		136	(133-140)		137	(134-141)		0.30
Sweet snacks	31	(29-32)		31	(30-32)		31	(30-33)		0.74	45	(44-47)	a	41	(40-42)	b	41	(40-42)	b	<0.01
Salty snacks	6.2	(6.0-6.4)		6.4	(6.2-6.6)		6.5	(6.1-6.9)		0.40	8.5	(8.1-8.9)	a	8.1	(7.9-8.3)		7.8	(7.4-8.2)	b	0.03

* : *P* value was computed based on F value for the main effect of the rationality and anti-emotionality personality group.

a, b, and c indicate a significant difference between values (i.e., a > b > c, *P* < 0.05), according to the post hoc test.

Consumption was adjusted for age, body mass index, physical activity, years of education, smoking habits, medical history, and total energy intake.

With regard to nutrient intake, as compared to participants in the lower level, both males and females in the higher level of the R/A-personality scale consumed more of the following foods (values in the parentheses are adjusted means in the case of males and females, respectively): protein (96 and 84 g), calcium (736 and 735 mg), iron (14 and 13 mg), carotene (3925 and 4204 µg), vitamin E (9 and 8 mg), vitamin C (121 and 125 mg), and dietary fiber (16 and 16 g). The corresponding nutrient values for males and females in the lower level of the R/A-personality scale were: 93 and 81 g, 696 and 700 mg, 13 and 12 mg, 3558 and 3771 µg, 8 and 8 mg, 109 and 116 mg, and 14 and 15 g, respectively (*P* < 0.01). Participants in the higher level of the R/A-personality scale consumed 2.8-11.5% more of the abovementioned nutrients than that consumed by participants in the lower level. Furthermore, both males and females in the higher level of the R/A-personality scale consumed more fat (62 and 56 g) and cholesterol (358 and 311 mg) than that consumed by participants in the lower level. The corresponding nutrient values for those in the lower level of the R/A-personality scale were: 60 and 56 g and 350 and 302 mg, respectively (*P* < 0.05). No difference in the consumption of salt was found among the 3 R/A-personality groups.

Table 3 shows the adjusted means of alcohol consumption. Males in the lower level of the R/A-personality scale drank 10.8% more alcoholic beverages, including sake, beer, and whisky, than those in the higher level did.

**Table 3.** Adjusted mean (95% confidence interval [CI]) of alcohol consumption (grams of pure ethanol per day) by the rationality/anti-emotionality personality group.

Item	Males										Females						
	Rationality/anti-emotionality score										Rationality/anti-emotionality score						
	Lower (0-5)	(95% CI)		Middle (6-8)	(95% CI)		Higher (9-11)	(95% CI)		P^*^	Lower (0-5)	(95% CI)	Middle (6-8)	(95% CI)	Higher (9-11)	(95% CI)	P^*^
Sake (Japanese rice alcohol)	14.852	(14.2-15.5)	a	14.141	(13.7-14.6)		13.509	(12.9-14.1)	b	<0.01	1.58	(1.4-1.7)	1.58	(1.4-1.7)	1.501	(1.3-1.7)	0.91
Beer	11.771	(11.5-12.1)	a	10.665	(10.4-11.0)	b	10.428	(10.1-10.7)	b	<0.01	3.002	(2.8-3.2)	2.844	(2.7-3.0)	2.765	(2.6-2.9)	0.39
Wine	0.395	(0.3-0.4)		0.316	(0.3-0.4)		0.395	(0.3-0.4)		0.27	0.237	(0.2-0.3)	0.316	(0.3-0.3)	0.237	(0.2-0.3)	0.73
Whisky	2.449	(2.3-2.6)	a	1.975	(1.8-2.1)	b	1.975	(1.8-2.1)	b	<0.01	0.632	(0.5-0.8)	0.553	(0.4-0.7)	0.553	(0.4-0.7)	0.54
Total alcohol consumption	34.918	(34.0-35.8)	a	31.521	(30.6-32.5)	b	30.81	(29.7-31.9)	b	<0.01	6.32	(5.9-6.8)	6.241	(5.9-6.6)	6.083	(5.8-6.4)	0.78

* : *P* value was computed based on the F value for the main effect of the rationality/anti-emotionality personality group.

a and b indicate a significant difference between values (i.e., a > b, *P* < 0.05), according to the post hoc test.

Consumption was adjusted for age, body mass index, physical activity, years of education, smoking habits, medical history, and total energy intake.

A total of 9.0% male participants and 33.5% female participants did not consume alcoholic beverages (participants who answered “not at all consume alcohol”).

## DISCUSSION

Both males and females with high R/A-personality scores showed higher vegetable consumption, including that of soy products and seaweed, than that shown by participants with low R/A-personality scores. Females with high R/A-personality scores showed higher fish and shellfish consumption than that shown by females with low R/A-personality scores. Males with high R/A-personality scores showed lower alcohol consumption than that of males with low R/A-personality scores. Snacking on sweet and salty foods was also reduced in females with high R/A-personality scores compared with that in females with low R/A-personality scores. Accordingly, males and females with high R/A-personality scores showed higher consumption of a variety of nutrients, including protein, calcium, iron, carotene, vitamin E, vitamin C, and dietary fiber than that in participants with low R/A-personality scores.

These results support previous Japanese studies,^22^^,^^23^ which suggested that individuals who show high degrees of emotional repression and rational thought are at reduced risk for disease. It appears that in Japan at least, an individual with a rational and unemotional personality may not be cancer-prone. For instance, soy products are reputed to be associated with a lower risk for certain cancers, cardiovascular diseases, and other adverse health outcomes,^30^^-^^32^ and they are also reported to prevent hot flashes and other menopausal symptoms in Japanese females.^41^^,^^42^ High consumption of green, yellow, and other vegetables is also associated with reduced risks of cardiovascular diseases and many cancers.^43^^-^^47^ Seaweed consumption, being particular to Japanese culture, appears to be linked to the prevention of cancers, including breast cancer,^48^^,^^49^ and cardiovascular diseases.^50^ Fish consumption has been demonstrated to have a protective effect on mortality from cardiovascular diseases^51^ and colorectal cancer, which is predominantly seen in females.^52^ Alcohol consumption, on the other hand, is reportedly associated with a higher risk for certain cancers and cardiovascular diseases.^53^^-^^55^

In the present study, males in the higher level of the R/A-personality scale consumed more meat and meat products and milk and dairy products, and females in the higher level of the R/A-personality scale consumed more eggs than that consumed by participants in the lower level of the R/A-personality scale. Participants in the higher level of the R/A-personality scale showed higher cholesterol and fat intake than those in the lower level of the R/A-personality scale did. To our knowledge, no study has found an association between egg consumption and coronary heart disease, even though egg consumption increases total serum cholesterol concentrations.^56^ Although meat and dairy products are considered risk factors for cancer and cardiovascular diseases, the results obtained are inconsistent.^57^^-^^59^ Further studies on personality characteristics and their relationship to dietary habits are required to confirm these findings.

Japanese culture is characterized by a strong emphasis on harmony and the suppression of individual emotion.^60^ In Japanese culture, there are negative connotations associated with the expression of emotion and lack of empathy. The expression of emotions focused on internal attributes such as anger and frustration is reinforced more in Western societies.^61^ Personality traits may be a reflection of cultural differences, which may influence social behavior, emotion, and health. Maladaptation to a cultural environment may undermine health-oriented dietary habits, and the expression of anger and hostility in interpersonal communication may cause mental distress, possibly resulting in an increase in alcohol intake, especially in males. In this study, mental distress and other psychological factors were not measured, and the existence of a mechanism linking personality characteristics and dietary habits could not be confirmed.

The limitations of this study were as follows: First, because tools for the measurement of personality had not been sufficiently validated when the present study was conducted, there was no standard available against which the R/A personality scale could be measured. In addition, although a certain degree of measurement error might have occurred while calculating the R/A personality score in our cohort, the factorial validity of this scale was confirmed in a previous study.^23^ In this study, the R/A-personality score was positively correlated with age. A Japanese study reported that anger expression decreased with increasing age.^62^ A tendency toward repressing emotion may be related to aging, and repressed emotion among the older population does not seem to be an exclusive characteristic of the Takayama cohort. It remains unclear whether the R/A-personality score changes with age, or whether generational differences are responsible for the variances in the scores. Second, the present study was conducted in 1992, and the dietary habits of Japanese people may have changed since that time. Nevertheless, according to the National Nutrition Survey by the Ministry of Health, Labour and Welfare,^63^ the trends in Japanese nutritional intake during the past decade have not dramatically changed. Third, even though the data in the current study were obtained from a large community sample, and the reliability and validity of food consumption has been well studied,^29^ due to the fact that the R/A-personality score was positively correlated with age, lapses in time should not be ignored. In addition, there could be a recall bias regarding food consumption that may have affected the R/A-personality score. For example, participants in the higher level of the R/A-personality scale tended to remember what they ate during the past year; therefore, their responses showed a higher consumption of a variety of foods than that observed in the case of participants in the lower levels of the R/A-personality scale. However, male consumption of alcoholic beverages and female consumption of sweet and salty snacks were higher in participants in the lower level of the R/A-personality scale than in the other groups.

The results of the present study suggest that psychological personality traits are related to dietary habits. Both males and females in the higher level of the R/A-personality scale consumed more soy products, green and yellow vegetables, other vegetables, and seaweed. Males in the higher level consumed more meat and dairy products and fewer alcoholic beverages, and females consumed more fish, shellfish, and eggs and fewer sweet and salty snacks than those in the lower level of the R/A-personality scale did. Whether psychosocial factors play a role in the prevention of diseases is an issue that should be explored in future studies.

## Appendix. Rationality/anti-emotionality personality questions.

**Table:**

1.	Do you always try to do things reasonably?
2.	Do you always try to understand other people?
3.	Do you try to behave rationally in the arena of human relationships?
4.	Do you try not to express emotions in the arena of human relationships?
5.	Do you try not to blame, criticize, or get angry at someone who condemns you?
6.	Can you always get on with relationships by repressing your emotion?
7.	Do you try to understand someone whose behavior is contradictory to your wishes?
8.	Does your behavior often change on an impulse? ^*^
9.	Do you often condemn others for being emotional? ^*^
10.	Do you try to understand someone whom you do not like?
11.	Do you think rationally and try not to criticize others whom you think are mean?

Participants were instructed to answer either “yes” (score 1) or “no” (score 0).

^*^ : Reversed item. If the participants answered “yes,” then they scored 0, and if they answered “no,” they scored 1.
